# A Review of Visual Representations of Physiologic Data

**DOI:** 10.2196/medinform.5186

**Published:** 2016-11-21

**Authors:** Rishikesan Kamaleswaran, Carolyn McGregor

**Affiliations:** ^1^Center for Biomedical InformaticsDepartment of PediatricsUniversity of Tennessee Health Science CenterMemphis, TNUnited States; ^2^University of Ontario Institute of TechnologyOshawa, ONCanada

**Keywords:** survey, human-centered computing, visualization application domains, information visualization, visualization systems and tools, visualization toolkits

## Abstract

**Background:**

Physiological data is derived from electrodes attached directly to patients. Modern patient monitors are capable of sampling data at frequencies in the range of several million bits every hour. Hence the potential for cognitive threat arising from information overload and diminished situational awareness becomes increasingly relevant. A systematic review was conducted to identify novel visual representations of physiologic data that address cognitive, analytic, and monitoring requirements in critical care environments.

**Objective:**

The aims of this review were to identify knowledge pertaining to (1) support for conveying event information via tri-event parameters; (2) identification of the use of visual variables across all physiologic representations; (3) aspects of effective design principles and methodology; (4) frequency of expert consultations; (5) support for user engagement and identifying heuristics for future developments.

**Methods:**

A review was completed of papers published as of August 2016. Titles were first collected and analyzed using an inclusion criteria. Abstracts resulting from the first pass were then analyzed to produce a final set of full papers. Each full paper was passed through a data extraction form eliciting data for comparative analysis.

**Results:**

In total, 39 full papers met all criteria and were selected for full review. Results revealed great diversity in visual representations of physiological data. Visual representations spanned 4 groups including tabular, graph-based, object-based, and metaphoric displays. The metaphoric display was the most popular (n=19), followed by waveform displays typical to the single-sensor-single-indicator paradigm (n=18), and finally object displays (n=9) that utilized spatiotemporal elements to highlight changes in physiologic status. Results obtained from experiments and evaluations suggest specifics related to the optimal use of visual variables, such as color, shape, size, and texture have not been fully understood. Relationships between outcomes and the users’ involvement in the design process also require further investigation. A very limited subset of visual representations (n=3) support interactive functionality for basic analysis, while only one display allows the user to perform analysis including more than one patient.

**Conclusions:**

Results from the review suggest positive outcomes when visual representations extend beyond the typical waveform displays; however, there remain numerous challenges. In particular, the challenge of extensibility limits their applicability to certain subsets or locations, challenge of interoperability limits its expressiveness beyond physiologic data, and finally the challenge of instantaneity limits the extent of interactive user engagement.

## Introduction

Two less formal reviews and one systematic review were published in the last decade, reporting positive impact of visual representations in the critical care setting. Sanderson et al provide a forward-looking analysis of representation of physiological data [1] in anesthesiology [2]. Drews and Westenskow review several graphical displays that facilitate rapid translation of physiological event knowledge for anesthesiologists [3]. An initial systematic review was published in 2007 by Görges and Staggers that reviews general physiologic data displays; however, with emphasis on surgical and anesthesiology specialities [4]. While those reviews provide important knowledge about the state of the art in physiologic data, they present only a partial aggregation of results, and limited knowledge that could be used to enhance the design of physiological visualizations. Furthermore, key elements such as the nature of visual variables utilized in the encoding, support for interactive exploration, and common design considerations were not discussed. All reviews focused on displays that support short-term patient monitoring tasks. Visualizations supporting longitudinal monitoring and interactive visual analysis of physiological data were not sufficiently addressed.

The aim of this specific review is of 3 parts: (1) identify the design decisions used in the development of novel physiologic visual representations; (2) review the utilization of temporal parameters namely: trajectory, frequency, and duration in visual designs using physiologic parameters; and (3) review the nature of interactive functions afforded for rich exploration tasks. With that in mind, this paper presents an analysis of a broad spectrum of physiological visual representations used at the bed-side, in the surgical ward, and for clinical research.

## Methods

The review was conducted in 2 phases: the first phase identified the key terms to be included in the search strategy, while the second phase broadened the search strategy and used structured analysis method. In the first phase we used Google Scholar, and 25 papers were found to be relevant. The search was limited to the last 15 years and used a combination of keywords that were known to the author, such as “(physiologic* or clinical or hemodynamic) and (visual* or graphic*) and (interface or display),” where asterisk was used to search for terms that started with the specific key words. In the second phase, we used 6 prominent sources including: IEEE Explore, ACM Digital Library, MEDLINE, EMBASE, ISI Web of Science, and Google Scholar. A broad search strategy was used to capture as many representations as were possible. Index terms were used to filter articles and included “data display*,” “diagnosis, computer-assisted,” “monitoring, physiologic/methods*,” “*computer graphics,” “user-computer interface,” “data display,” “interview* or discussion* or questionnaire* or “focus group*” or qualitative or ethnograph* or fieldwork or “field work” or “key informant,” “task performance and analysis,” “graphic* adj2 display*”.

For screening articles in the second phase, we used rigorous inclusion criteria (Textbox 1) that initially classified visualizations across 4 groups. The groups were (1) tabular displays, (2) waveform displays, (3) object displays, and (4) ecological displays. Inclusion criteria relating to outcome measures are divided into 3 sets of measures (Textbox 2). They include temporal and duration, human and qualitative factors, and quantitative measures. The physiological parameters tested are listed in Textbox 3. We placed a restriction in years from January 1, 1983 to August 1, 2016 and limited our results to human studies in critical care, anesthesiology, and surgery. We included snowballing of references and manual searches on Google Scholar and PubMed. This resulted in a total of 1262 titles generated for review. Relevant titles were identified using rigorous inclusion criteria (Textbox 1). In total, 171 titles were then designated for abstract review. Following that, 78 abstracts were selected for full review, and 39 papers were selected for inclusion in the analysis. Bias was mitigated by having 2 researchers screen independently, and differences were resolved through discussions until consensus was reached.

**Inclusion criteria.**

Types of studies:

• Randomized controlled trials, cohort, case-control, and design studies.

• The review placed increasing preference for randomized control trials, followed by cohort, case-control, and finally design studies. Design studies are popular in the visualization community and were included to study results pertaining to user-evaluations.

Types of participants:

• Critical care nurses and physicians.

• Several studies have only tested interventions on physicians and excluded nurses, while other studies have used naive participants usually by recruiting undergraduates.

Types of interventions:

• Novel knowledge representations, numeric, waveform or metaphor-based displays.

• We focus on the intervention in which physiological display is not represented exclusively in waveform and/or static numerical forms.

**Reported metrics.**

Temporal metrics:

• Time to detection of adverse event(s), time to diagnose(s), time to initiate treatment(s)

Human factors:

• NASA-TLX task load index score, satisfaction of intervention (Likert scales), number of participants, clinical expertise of participants, setting in which the trials were conducted, noise level of the environment, age of the participants, caffeine intake

Clinical relevance:

• Accuracy of diagnoses, accuracy of treatment

**Physiological parameters tested.**

Physiological parameters:

• Central venous pressure (mm Hg)

• Mean left arterial pressure (mm Hg)

• Systemic vascular resistance

• ST segment depression of ECG (mm)

• Arterial oxygen saturation (%)

• Heart rate (bpm)

• Respiratory wave (impedance)

• End-tidal CO2

• Mean arterial blood pressure (mm Hg)

• Pulmonary vascular resistance

• Cardiac output (mL/min)

• Stroke volume (mL)

• Peripheral oxygen saturation (%)

• Respiratory rate (rpm)

• Pulse rate

• Mean pulmonary artery pressure (mm Hg)

Following the creation of the inclusion criteria, an online data extraction form was developed using Google forms and used to evaluate all papers. The data extraction form consisted of 6 sections that were identified as potential areas of interest. For each full paper reviewed, 74 questions were screened. Questions to be included in the data extraction forms were selected from themes identified in the pilot study. In particular, questions were generated to elicit detail about the study, design, and results from any human experiment or evaluations. Where appropriate the questions were marked as either not reported if data was missing, or not applicable if the question was a follow-up of a prior conditional question. The data was then thematically synthesized based on aggregations of results by descriptive codes. The thematic synthesis is presented using a series of matrices presented in the next section.

## Results

### Phase 1 and 2

**Figure 1:**
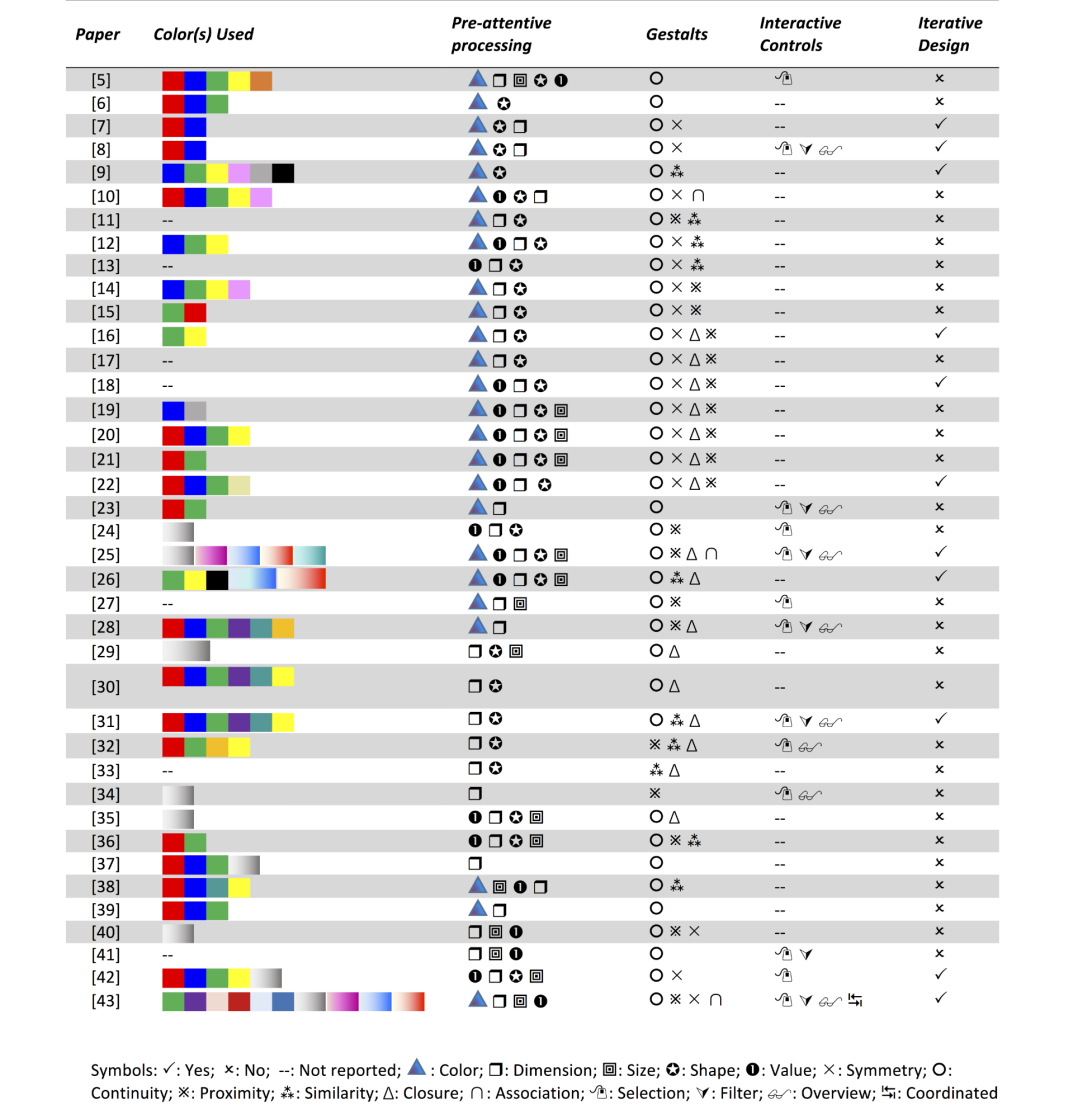
Comprehensive Matrix of Design Properties.

All papers included in the analysis were passed through the data extraction form and resulted in an initial comprehensive matrix of results. Of 74 questions that were initially probed, results that yielded over 75% not reported, or not applicable across all papers analyzed were removed from our analysis. Then 39 variables were selected for inclusion in the initial matrix. Phase 1 results are summarized in the comprehensive matrix of design properties (Table 1 and Figure 1) and phase 2 results are summarized in the Comprehensive Matrix of Study Results (Table 2 and Table 3). The comprehensive matrix of design properties presents 10 variables which are divided between 2 tables. Variables appearing in (Table 1) are “Target Users”, “Year”, “Clinical Context”, “Number of Variables”, and “Display Type”.

“Target Users” relates to the clinical specialty, and “Year” is the approximate date the prototype was tested. Due to the difference between the dates of publication and evaluation, this value was approximated based on the date of submission of the article. “Clinical Context” conveys the copresence of contextual clinical information, and “Number of Variables” refers to the total number of physiological or clinical variables that were visible in a single screen. “Display Type” lists the types of graphics utilized by the paper belonging to one of: tabular, object, or metaphoric displays. “Color(s) Used” identifies the hue where available. “Pre-attentive Processing” lists particular visual variables that were used in the visual representation such as: shape, size, and dimension. “Gestalts” refers to the designer’s use of grouping laws identified by Gestalt’s laws of perception: the use of proximity, similarity, closure, symmetry, and continuity as a means of discerning visual objects presented in the display [5]. Finally “Interactive Controls” refers to the ability for the display to support direct manipulation of one or more properties and “Iterative Design” identifies displays that were built using user-centered design approaches that include users into key decision making processes prior to the development of the display.

A second matrix, titled the Comprehensive Matrix of Study Results presents additional 11 variables that were identified in papers which presented study results. Table 2 lists “Setting,” “Study Type,” “Results Reported,” “Realism,” “Cognitive Workload,” “Historic Trends,” “Visual Encoding for Temporal Trajectory,” “Visual Encoding for Duration,” “Visual Encoding for Frequency”. Table 3 lists “Counter-balanced for Display or Scenario,” “Were Scenarios Clinically Relevant,” and “Function Supporting Case-controlled Analysis.” “Setting” describes the location where the study was physically conducted; for instance, the lab, clinic, or public areas. “Study Type” identifies research method used to validate the display. “Results Reported” summarizes key findings from the study, and “Realism” presents the latency of the display as well as the ability of the display to mimic real-world dynamism. “Cognitive Workload” reports on findings indicating reduced or increased workload and “Historic Trends” identifies displays that present historical trends that are greater than 5 minutes. The variables beginning with visual encodings for temporal trajectory, duration, and frequency identify particular techniques used by the displays to represent trends, duration of events, and frequency of events. The counter-balanced variable identifies methodologies that used strategies to minimize learning effects during the experiment. Finally, the clinical scenario variable lists the displays that were evaluated using real-world clinical scenarios. These tables, along with descriptions of the results are presented in the next section.

### Comprehensive Matrix of Design Properties

The goal of the comprehensive matrix of design properties is to present design decisions that were followed to develop prototypes across all 39 papers analyzed. Visual representations were found across mainly anesthesiology (n=17), critical care (n=20), and in some multi-discipline (n=2) environments. Only one display was developed as a tool for intensive nurses [42]. Multi-discipline environments consist of 2 or more specialities, such as integrated in-patient and out-patient systems. Visual displays started to become actively contributed from the early 1990s, then increasing every 10 years, 1984 (n=1), 1985-1994 (n=8), 1995-2004 (n=13), and 2005-2016 (n=17). Integrated clinical data was also found across some displays (n=16), while a greater number of displays were devoted to the display of physiological waveforms (n=24). Number of variables presented in a single screen was wide-ranging; most displays contained greater than 20 variables per screen (n=15), followed by 11-20 variables (n=12), while 7 displays contained between 0-4 variables.

Reviewed visual representations included a mix of display formats, such as tabular (TB), object-based (OB), waveform (WF), and metaphoric (MT). Standalone MT representations were most commonly seen (n=12), followed by standalone WF (n=6). With respect to combinatory displays, TB appeared with WF (n=6) most frequently, WF with MT (n=6), and followed by WF with an OB (n=4). When identifying the display type most frequently paired in a combinatory display, WF (n=12) appeared most often, followed by TB (n=7) and OB (n=6) displays. Overall, across all identified papers including those where multiple representatives were presented, metaphoric displays were the most popular (n=22), followed by waveform displays (n=20), and object displays (n=10).

Visual representations utilized at least 2 of the primary colors, red, blue or green (n=21), while yellow (n=11) and turquoise (n=4) were also popular options. Three papers utilized discrete color encoding, 2 papers [25,43] mentioned the source of their color coding. A number of papers did not specify the type of color that was used (n=10). Pre-attentive processing of items were commonly exploited through manipulating visual variables such as color (n=24) and size (n=12), followed by dimension (n=7), and shape (n=5).

Visual representations also exploited some aspect of Gestalt’s law of groupings, such as continuity (n=18) with waveform displays, closure (n=17) when identifying boundaries, symmetry (n=14) with visual metaphors and object-based displays, and proximity (n=7) to aid in higher level detection of abnormal events. The most popular interaction method that was supported was selection (n=13). Selection allows the user to select visual objects directly to reveal greater details. This was followed by interactive filtering (n=7) to select partial ranges such as short durations of time. Finally, in many cases designs were proposed without following user-centered design approaches (n=28). In total, 10 papers reported using user-centered design processes, while 4 papers described a structured approach used in developing the proposed visual design [5,31,43,44].

**Table 1:** Comprehensive matrix of design properties.

Paper	Target users	Clinical context	Number of variables	Display type
Engelman et al, 2014 [6]	Intensivists	Yes	>20	Waveform display (WF)
Charabati et al, 2009 [7]	Anesthesia	No	0-4	WF
Agutter et al, 2003 [5]	Anesthesia	No	11-20	Metaphoric display (MT)
Anders et al, 2012 [8]	Intensivists	Yes	>20	WF, MT
Wachter et al, 2004 [9]	Intensivists	No	5-10	MT
van Amsterdam et al, 2013 [10]	Anesthesia	No	5-10	MT
Kennedy et al, 2011 [11]	Anesthesia	No	0-4	Object-based display (OB)
Liu et al, 2005 [12]	Intensivists	No	5-10	MT
Blikeet al, 1999 [13]	Anesthesia	No	11-20	OB
Cole et al, 1994 [14]	Intensivists	No	5-10	MT
Deneault et al, 1990 [15]	Anesthesia	No	5-10	MT
Jungk et al, 2000 [16]	Anesthesia	No	>20	OB, MT
Gurushanthaiah et al, 1995 [17]	Anesthesia	No	5-10	WF, MT
Ireland et al, 1997 [18]	Intensivists	Yes	>20	MT
Tappan et al, 2009 [19]	Anesthesia	No	5-10	MT
Michels et al, 1997 [20]	Anesthesia	No	>20	MT
Effken et al, 1997 [21]	Intensivists	No	5-10	OB, MT
Görges et al, 2012 [22]	Intensivists	Yes	11-20	WF, MT
Stylianides et al, 2011 [23]	Intensivists	Yes	>20	WF
Litt et al, 1992 [24]	Intensivists	Yes	>20	WF, MT
Gschwandtner et al, 2011 [25]	Intensivists	Yes	>20	WF, MT
Horn et al, 2001 [26]	Intensivists	Yes	11-20	MT
Dayhoff et al, 1994 [27]	Intensivists	Yes	>20	WF
Norris et al, 2002 [28]	Intensivists	Yes	>20	Tabular display (TB), WF
Langner, 1952 [29]	Intensivists	No	0-4	WF
Burykin et al, 2011 [30]	Intensivists	Yes	0-4	WF
Miller et al, 2009 [31]	Intensivists	Yes	>20	TB, WF, OB
Kruger et al, 2011 [32]	Anesthesia	Yes	11-20	MT
Law et al, 2004 [33]	Intensivists	No	5-10	TB, WF
Ahmed et al, 2011 [34]	Intensivists	Yes	>20	TB
Sainsbury, 1993 [35]	Anesthesia	No	11-20	WF, OB
Zhang et al, 2002 [36]	Anesthesia	No	5-10	OB, MT
Kennedy et al, 2008 [37]	Anesthesia	No	0-4	WF
Lowe et al, 2001 [38]	Anesthesia	No	0-4	OB
Charbonnier, 2004 [39]	Intensivists	No	0-4	TB, WF
Shabot et al, 1986 [40]	Anesthesia	No	>20	TB, MT
Eden et al, 2006 [41]	Anesthesia	Yes	>20	TB, WF, OB
Koch et al, 2013 [42]	Nurses	Yes	>20	TB, WF, MT
Kamaleswaran et al, 2016 [43]	Intensivists	Yes	11-20	WF, OB, MT

**Table 2:** Comprehensive matrix of study results.

Paper	Setting	Study type	Results reported	Realism	Cognitive workload	Historic trends	Visual encoding for temporal trajectory	Visual encoding for duration	Visual encoding for frequency
Engelman et al, 2014 [6]	ICU	Eval.^a^	Pos.^b^	Live	–	C^c^	C	–	–
Charabati et al, 2009 [7]	Lab	Exp.^d^	Pos.	Static	↓	–	C	–	–
Agutter et al, 2003 [5]	Lab	Exp.	Pos.	Sim.^e^	↓	–	–	–	–
Anders et al, 2012 [8]	ICU	Exp.	±	Static	0^f^	C	C	–	–
Wachter et al, 2004 [9]	ICU	Eval.	Pos.	Live	–	–	G^g^	–	–
van Amsterdam et al, 2013 [10]	Lab	Exp.	Neg.^h^	Static	–	–	O^i^	–	–
Kennedy et al, 2011 [11]	Lab	Exp.	Pos.	Sim.	–	–	O	–	–
Liu et al, 2005 [12]	Lab	Exp.	Pos.	Sim.	–	–	–	–	–
Blikeet al, 1999 [13]	Lab	Exp.	Pos.	Sim.	–	–	–	–	–
Cole et al, 1994 [14]	Lab	Exp.	Pos.	Static	–	–	G	G	G
Deneault et al, 1990 [15]	Lab	Exp.	Pos.	Sim.	–	–	–	–	–
Jungk et al, 2000 [16]	Lab	Exp.	Pos.	Sim.	–	–	C	–	–
Gurushanthaiah et al, 1995 [17]	Lab	Exp.	Pos.	Sim.	–	–	C	–	–
Ireland et al, 1997 [18]	Lab	Eval.	Pos.	Static	–	–	C, G	–	–
Tappan et al, 2009 [19]	Lab	Exp.	Pos.	Sim.	↓	–	C, G	–	–
Michels et al, 1997 [20]	Lab	Exp.	Pos.	Sim.	–	–	–	–	–
Effken et al, 1997 [21]	Lab	Exp.	Pos.	Sim.	–	–	–	–	–
Görges et al, 2012 [22]	ICU	Exp.	Pos.	Sim.	0	–	G	G	G
Stylianides et al, 2011 [23]	ICU	Eval.	Pos.	Live	–	C	C	–	–
Litt et al, 1992 [24]	Lab	App.^j^	–	Static	–	C	C	G	G
Gschwandtner et al, 2011 [25]	Lab	Des.^k^	–	Static	–	C	C	C	–
Horn et al, 2001 [26]	ICU	Eval.	Pos.	Static	↓	C	G	G	–
Dayhoff et al, 1994 [27]	ICU	App.	–	Live	–	–	C	–	–
Norris et al, 2002 [28]	ICU	App.	Pos.	Live	–	C	C	–	–
Langner, 1952 [29]	ICU	Eval.	–	Static	–	–	C	–	–
Burykin et al, 2011 [30]	ICU	App.	–	Sim.	–	–	C	–	–
Miller et al, 2009 [31]	ICU	Exp.	Pos.	Static	–	–	C	T^l^	T
Kruger et al, 2011 [32]	Surgery	App.	–	Live	–	–	G	T	T
Law et al, 2004 [33]	Lab	Exp.	–	Static	–	–	C, T	T	T
Ahmed et al, 2011 [34]	Lab	Exp.	Pos.	Sim.	↓	–	T	T	T
Sainsbury, 1993 [35]	Surgery	Eval.	Pos.	Live	–	–	C	–	–
Zhang et al, 2002 [36]	Lab	Exp.	±	Sim.	±	–	C, G	G	G
Kennedy et al, 2008 [37]	Lab	Exp.	Pos.	Sim.	–	–	C	–	–
Lowe et al, 2001 [38]	Lab	App.	Pos.	Sim.	–	–	C	–	–
Charbonnier, 2004 [39]	Lab	Des.	–	Sim.	–	–	C	–	–
Shabot et al, 1986 [40]	Lab	Des.	–	Sim.	–	C	C	–	–
Eden et al, 2006 [41]	Surgery	App.	Pos.	Live	↓	C	C	–	–
Koch et al, 2013 [42]	ICU	Exp.	Pos.	Sim.	↓	C	C	–	–
Kamaleswaran et al, 2016 [43]	ICU	Eval.	Pos.	Live	–	C, G	C, G, O	G, O	G, O

^a^Eval: Evaluation

^b^Pos: Positive

^c^C: Curves

^d^Exp: Experiment

^e^Sim: Simulated

^f^0: No Change

^g^G: Glyph

^h^Neg: Negative

^i^O: Object

^j^App: Application

^k^Des: Design

^l^T: Text

**Table 3:** Comprehensive matrix of study results.

Paper	Counter-balanced for display or scenario	Scenarios were clinically relevant	Function supporting case-controlled analysis
Engelman et al, 2014 [6]	–	Yes	–
Charabati et al, 2009 [7]	Display	Yes	–
Agutter et al, 2003 [5]	Display	Yes	–
Anders et al, 2012 [8]	Display & Scenario	Yes	–
Wachter et al, 2004 [9]	–	No	–
van Amsterdam et al, 2013 [10]	Display	Yes	–
Kennedy et al, 2011 [11]	Display	No	–
Liu et al, 2005 [12]	Display & Scenario	Yes	–
Blikeet al, 1999 [13]	Scenario	Yes	–
Cole et al, 1994 [14]	Display & Scenario	Yes	–
Deneault et al, 1990 [15]	Display & Scenario	Yes	–
Jungk et al, 2000 [16]	Scenario	Yes	–
Gurushanthaiah et al, 1995 [17]	Scenario	Yes	–
Ireland et al, 1997 [18]	–	No	–
Tappan et al, 2009 [19]	Display & Scenario	Yes	–
Michels et al, 1997 [20]	Display & Scenario	Yes	–
Effken et al, 1997 [21]	Scenario	Yes	–
Görges et al, 2012 [22]	Scenario	Yes	–
Stylianides et al, 2011 [23]	–	No	–
Litt et al, 1992 [24]	–	No	–
Gschwandtner et al, 2011 [25]	–	Yes	✓
Horn et al, 2001 [26]	–	No	–
Dayhoff et al, 1994 [27]	–	No	–
Norris et al, 2002 [28]	–	No	–
Langner, 1952 [29]	–	No	–
Burykin et al, 2011 [30]	–	No	–
Miller et al, 2009 [31]	Display & Scenario	Yes	–
Kruger et al, 2011 [32]	–	No	–
Law et al, 2004 [33]	Display & Scenario	Yes	–
Ahmed et al, 2011 [34]	Display & Scenario	Yes	–
Sainsbury, 1993 [35]	–	No	–
Zhang et al, 2002 [36]	Scenario	Yes	–
Kennedy et al, 2008 [37]	Display & Scenario	No	–
Lowe et al, 2001 [38]	–	No	–
Charbonnier, 2004 [39]	–	No	–
Shabot et al, 1986 [40]	–	No	–
Eden et al, 2006 [41]	–	No	–
Koch et al, 2013 [42]	Display & Scenario	Yes	–
Kamaleswaran et al, 2016 [43]	Display & Scenario	Yes	–

### Comprehensive Matrix of Study Design

**Figure 2:**
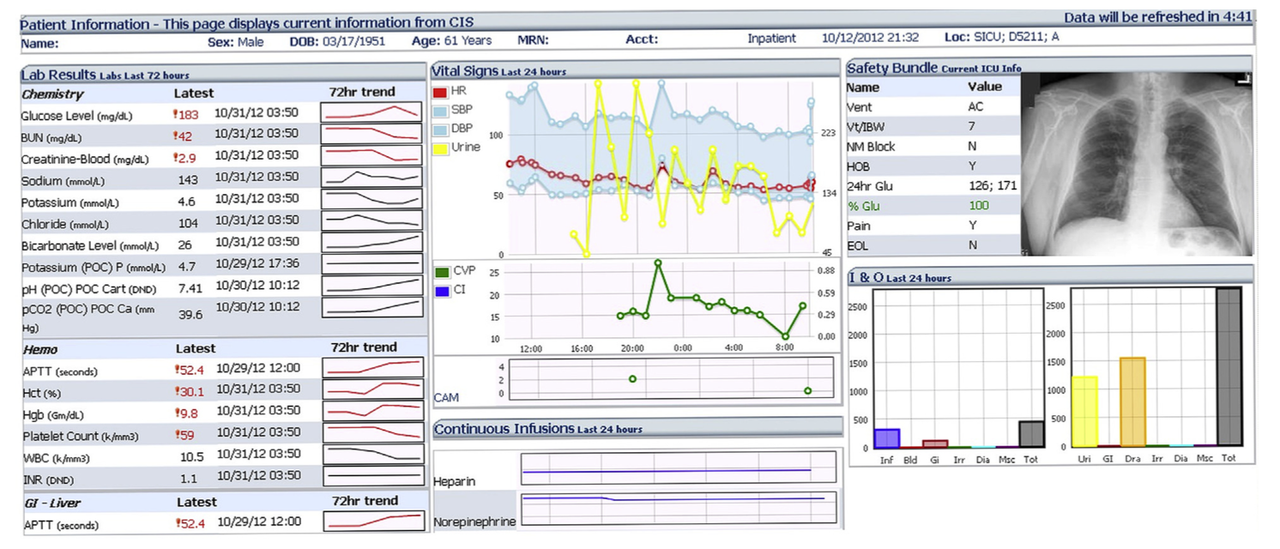
A tabular display that mimics traditional clinical flow-sheets.

The Comprehensive Matrix of Study Design (Figure 2) presents the results that were reported by authors for any evaluation or experiment. While the search strategy yielded 39 full papers that were identified for analysis, only 29 of these papers contained primary study results from a case study, evaluation, or human experiment, and employed 1 of naïve, novice, or expert participants in the evaluation method. Naïve participants were generally undergraduate students with little or no prior clinical knowledge. Novice participants ranged from undergraduate computer science or nursing students to newly graduated clinical staff. Expert participants had at least 10 years of experience.

The number of participants exposed to test conditions highly varied; however, the majority of studies employed at least 15 participants. Six studies used a sample size greater than 20 to test for detection, diagnostic, and treatment accuracy, with the minimum and maximum being 4 and 32 participants, respectively. Most displays integrated these systems in a single display using live or static representations (n=15), while displays that were presented as case studies (in situ) were connected to central monitoring systems. Some displays supported views of clinical information that integrated data from other clinical and laboratory systems (n=15) [45]. Most prototypes that were evaluated used more than one data stream, with the exception of the studies that contained low-frequency updates (n=9). Most evaluations or experiments utilized more than one condition to test each display; however, a few did not have any scenarios or patient conditions (n=9). A large number of studies also did not utilize data from more than one patient-source (n=26).

Most of the studies were conducted in laboratory environments (n=24), followed by evaluations or experiments in the intensive care unit (n=12). Some studies were evaluated over multiple specialities (n=2). A majority of studies used some form of experimentation to validate their designs (n=21), although the specific method of experimentation was not always explicitly mentioned. Evaluations involved clinicians and mixed qualitative and quantitative methods were used to report results (n=8). Applications were primarily qualitative in nature, often depicting results through anecdotes (n=7). The remaining studies were design papers that investigated novel visual representations without involving prototypes. Of the papers that reported results (n=31), most reported positive findings (n=27), but in some cases negative results were also reported (n=4). A between-group experimental study yielded site-dependent results that were skewed towards the site that produced the visual representation. For evaluations or experiments the source of data to support realism was spread across live simulations (n=19), live patient-origin data (n=9), or static patient-generated data (n=11). Most studies did not test for cognitive overload using ad hoc methods or traditional workload score metrics such as the NASA Task Load Index (NASA-TLX) (n=30). Where cognitive workload was reported (n=7), most were reported to have reduced cognitive overload (n=5), while others reported no change or mixed results (n=3).

Long-term historical values, specifically ranges exceeding 5 minutes of monitoring were not included in majority of the displays (n=28). Tri-event parameters, namely, trajectory, frequency, and duration were seldom supported by visual representations, where these parameters were identified, trajectory was most frequently found (n=27). Temporal trajectory was encoded using curves (n=25) such as in a line graph, or glyphs (n=9). In terms of duration, the second tri-event parameter was seen across 10 displays, of which, glyphs (n=6), text (n=4) or curves (n=2) representations were utilized. Frequency, the last tri-event parameter was also seen in some visual representations encoded by glyph (n=5) or text (n=4) where supported. Where displays were validated through experimentation, both the display and scenarios were more often counterbalanced (n=12), while some experiments counterbalanced only the scenario (n=6) and others only the display (n=4). Scenarios were utilized across many studies utilizing experimentation or evaluation methodologies (n=22) and most were clinically relevant problems (n=21). Finally, only one of the evaluated visual representations supported the ability to perform analysis across multiple patients.

## Discussion

### Principal Findings

A total of 19 novel visual representations were identified from analysis of the literature. Novel displays were seen across 4 main groups, including tabular, waveform (graph-based), object, and metaphors. The latter 2 are aggregated together as ecologic displays.

### Tabular Displays

The early- 1990’s saw growing interest in converting large volumes of paper patient charts to “virtual” records [24,46-49]. Initial representations adopted by these virtual patient records were largely tabular and text-dominant, and sometimes contributed negatively to information overload [48]. Figure 2 [50] illustrates an example of a traditional virtual patient chart that mimics a traditional paper flow chart. This review identified 14 tabular representations published from 1952 to 1997. Those systems provide a direct manipulation using the traditional desktop-oriented, Windows-Icon-Mouse-Pointer (WIMP) interaction paradigm. Additional levels of interactions, such as multiple mouse clicks, are required to access unique views of patient data. Large number of these displays are often duplicated to a physical copy, in part due to the simplicity and ease of reading paper charts [51].

**Figure 3:**
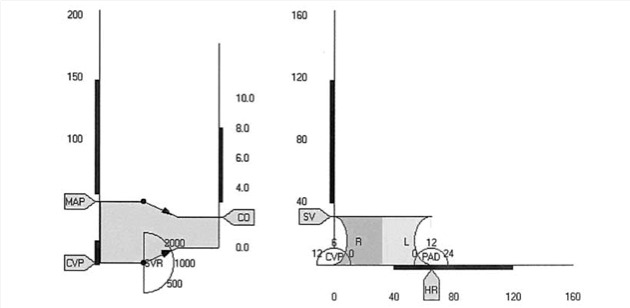
A modern dashboard utilizing waveform displays.

### Waveform Displays

The review identified 9 out of 39 studies that used some form of live physiologic streams from real patients to display largely identical waveform representations. It was also noted that much of these waveform displays were integrated with other tabular and text representations. Five papers that presented waveform displays also supported interactive capabilities, including the ability to select regions of interest, filter based on patients, and generate screen captures [6,8,25,43,52]. Stylianides and colleagues (2011) present an engine for producing waveform graphics [23]; however, their system serves the purpose of animating historic physiologic data streams. CareCruiser [25], supports the interactive exploration of treatment plans using physiologic data. However, that system was not evaluated using more than one clinical user. PhysioEx [43] was evaluated using an expert evaluation methodology employing 5 domain experts, and was shown to further enhance that interactive analytic workflow by providing coordinated analysis of temporal data streams; however, using waveform displays only to guide the user with additional context.

Despite their ability to communicate acute time-sensitive events [20], waveform representations have numerous limitations [4,31,53]. One prime disadvantage of waveform displays is the potential to negatively impact cognitive load, that is, they require humans to monitor and consume large numbers of data points as they are produced to derive trends and higher level knowledge [7,8,22]. These waveforms display can convey several features in one frame; therefore, easily disturb limited resources of the working memory capabilities [54]. The challenge of managing large volumes of data have been extensively studied in several domains, such as information overload [55], visual data mining [56], and addressing cognitive challenges related to interruptions, task performance, and decision making [55,57-59].

Integrated methods of representing critical physiological information have been actively studied to reduce the internal mental processing requirement [20,22,32,60-62]. These integrated displays use a combination of text [33,34], graphic [3,4,63], and waveform [64,65] representations to summarize low-level information. Figure 3 [6] illustrates an example of an integrated display. Three such integrated displays were identified in the review [6,8,25]. These displays support clinicians to interactively select regions of interest while monitoring other forms of slow-changing clinical data. However, only one display allows the clinician to compare against a cohort [25]. Other studies, seeking alternatives to the waveform visual encoding, propose novel and ecological methods to improve knowledge discovery and minimize cognitive overload.

### Ecological Displays

#### Classes of Visual Representations

Ecologic displays attempt to integrate relationships existing across both workflows and semantics [66]. Among the primary goals of ecologic displays is to convey both the means-end relation, answering the particular means of arriving at that state and its ultimate consequence. From our review, we identified 2 large classes of visual representations that approach these objectives. Object-oriented displays, and metaphoric displays were seen to extend typical limitations found in text, tabular, and waveform displays by introducing novel information, such as spatial and temporal arrangements of closely related information.

#### Object-Oriented Displays

Displays that utilize and manipulate 2-dimensional graphical objects, limited to basic shapes and symmetries to produce emergent properties have been classed as object-oriented displays [2,13,35]. These displays follow demonstrated efficacy of graphical displays over traditional numeric displays observed in nuclear power station control stations [67]. Studies have shown a positive relationship with integrated displays and an overall improvement in diagnosis ability as well as a reduction in time to initiate treatment [68].

**Figure 4:**
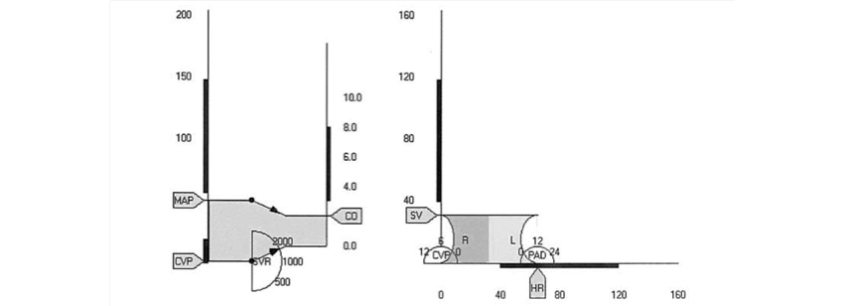
Advanced graphical display for hemodynamic monitoring.

Blike and colleagues (2000) [69] showed that subjects exposed to emergent features using novel graphics recognized a problem more rapidly, but their accuracy had not improved in comparison to the numeric display. Moreover, they showed that the shape of the graphic, illustrated in Figure 4 [13], improved detection of etiology compared to the numeric and control displays. While Blike and colleagues stated an improved reaction and fewer errors when using the object-oriented display, the display was found to be confusing and not ecological to naïve participants. Zhang and colleagues [36] reproduced the designs introduced by Blike et al, and found that anesthesiologists were able to detect simple deviations faster; however, no change was seen with detection times of more complex cardiovascular events. Other studies have reported similar conclusions [5,9,13,68,70], suggesting a link between detection and reactionary time to the format and features of the graphical display.

**Figure 5:**
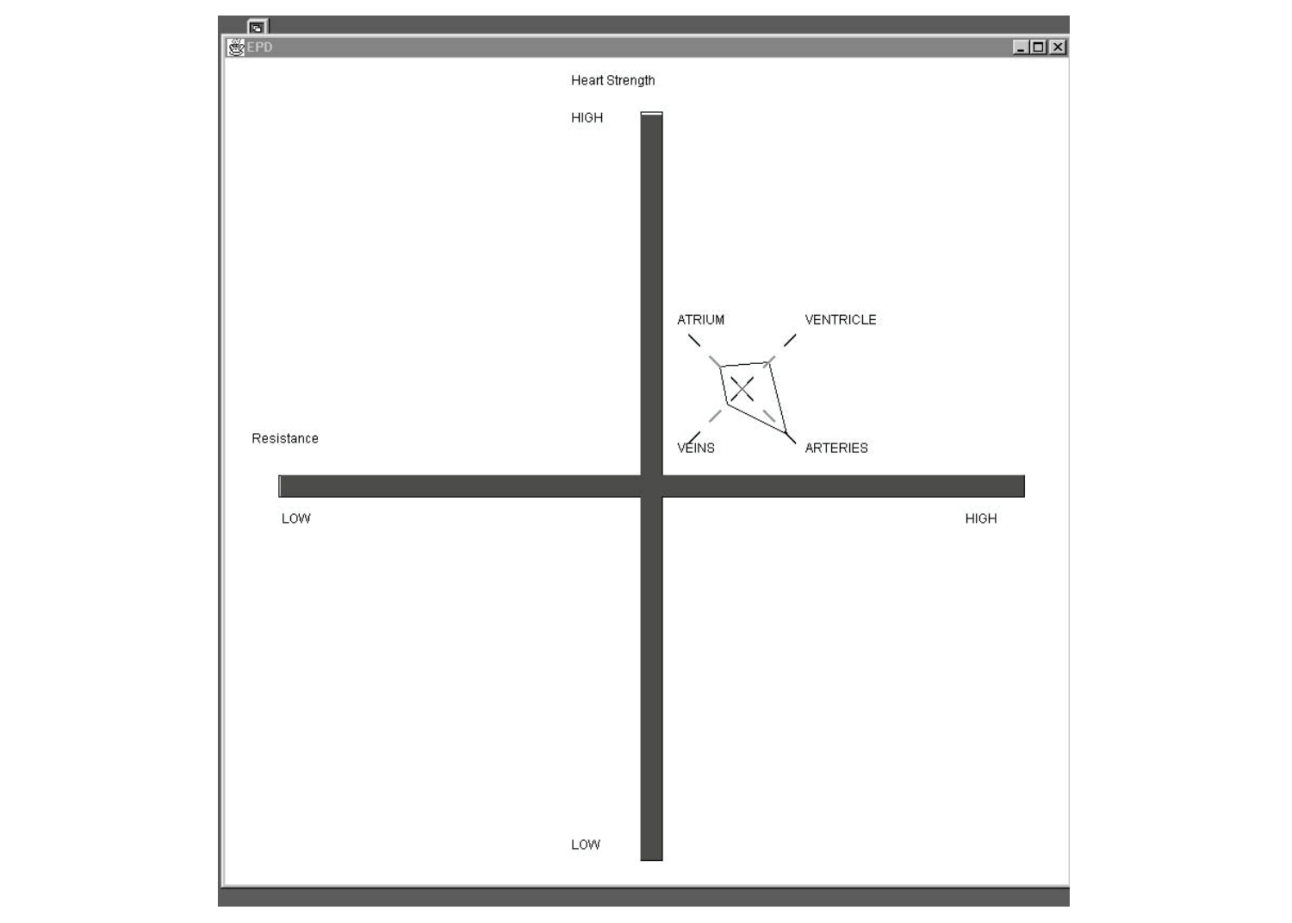
The etiological potential display moves an object across 4 quadrants of heart strength and resistance. The object in the top right quadrant is distorted to show relative depressions in the atrium, ventricles, veins and arteries.

In contrast, other studies that extrapolated heuristics from object-oriented displays report less convincing evidence; for instance, some report negative links when participants were presented object-oriented displays [21,37]. The etiological potential display (Figure 5) [21] attempts to extract specific features of object displays that improve detection and diagnosis. In that study, Effken and colleagues find no significance in the detection or diagnostic times, even when 3 abstract displays were tested. Two of these displays required that features of the full prototype either be reorganized or removed.

#### Metaphoric Displays

A total of 20 representations, for over half of all visual representations analyzed, belonged to the metaphoric display group. Most clinical metaphoric displays illustrate physiologic data in terms of organ-systems [20,36,44,71]. Five papers presented metaphors that involved dynamic objects that exhibited behaviors similar to organ systems [5,14,20,21,26].

**Figure 6:**
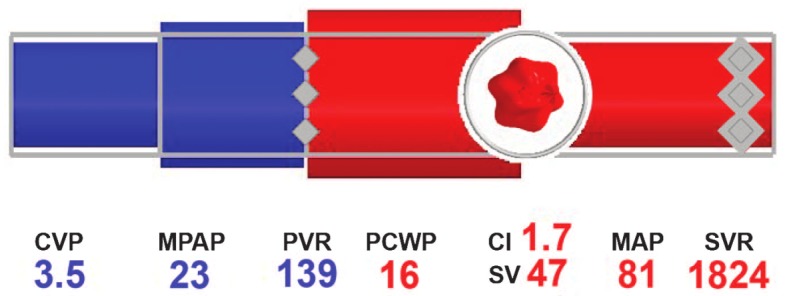
Volume triangles represent multivariate clinical data using a lung-expansion metaphor.

**Figure 7:**
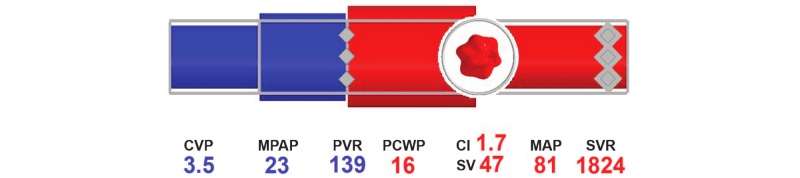
Graphical Cardiovascular Display, adapts a metaphor of a pipes with volume and pressure properties.

**Figure 8:**
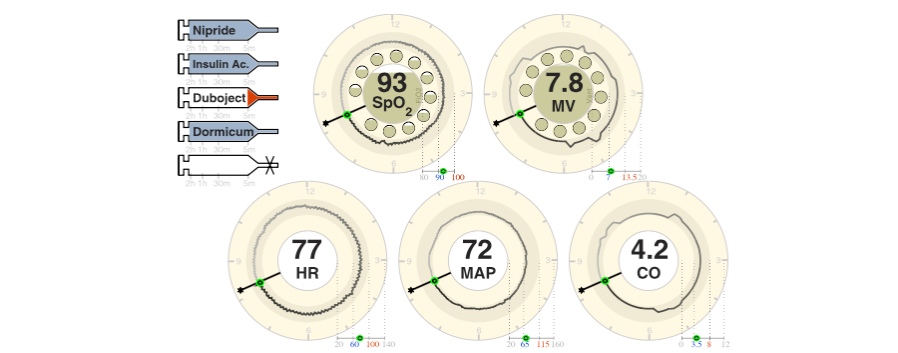
Far-view visual metaphors for triaging vital signs.

Several papers identified metaphor displays with positive outcomes. Cole and Stewart (1994) [14], introduced a visual representation (Figure 6) [50] which consists of 2 volume rectangles that compress or expand similar to the respiratory system. This design was further improved with additional data dimensions [26]. A Graphical Cardiovascular Display (Figure 7) [5] that uses a pipe-like metaphor of the cardiovascular system, was shown to enable faster detection of adverse events [5]. Wachter and colleagues (2003) applied similar approaches to develop a respiratory interface and found participants were able to identify abnormal states faster [9]. Gorges and colleagues, introduced a series of visual metaphors to communicate visual signs to bed-side clinicians [22]. These displays adopt a clock metaphor illustrated in Figure 8 [22] to convey salient features, such as temporal trends over the past 12-hours. Charabati and colleagues from the Montreal General Hospital’s department of anesthesiology introduced a gauge metaphor to highlight normal and abnormal ranges, and conducted an evaluation across 2 sites [7]. They found a combination of numeric and visual metaphors achieved the strongest advantage in detection, accuracy, and workload. Tappan and colleagues evaluated visual metaphors by appending visual objects to traditional medical monitors [19]. They reported significant improvements in detection of adverse events, with the visual metaphor having a 14.4 second advantage over traditional physiologic monitors. The visual metaphor was also found to reduce the number of missed events. However, similar to previous studies, these investigations were conducted in controlled environments.

**Figure 9:**
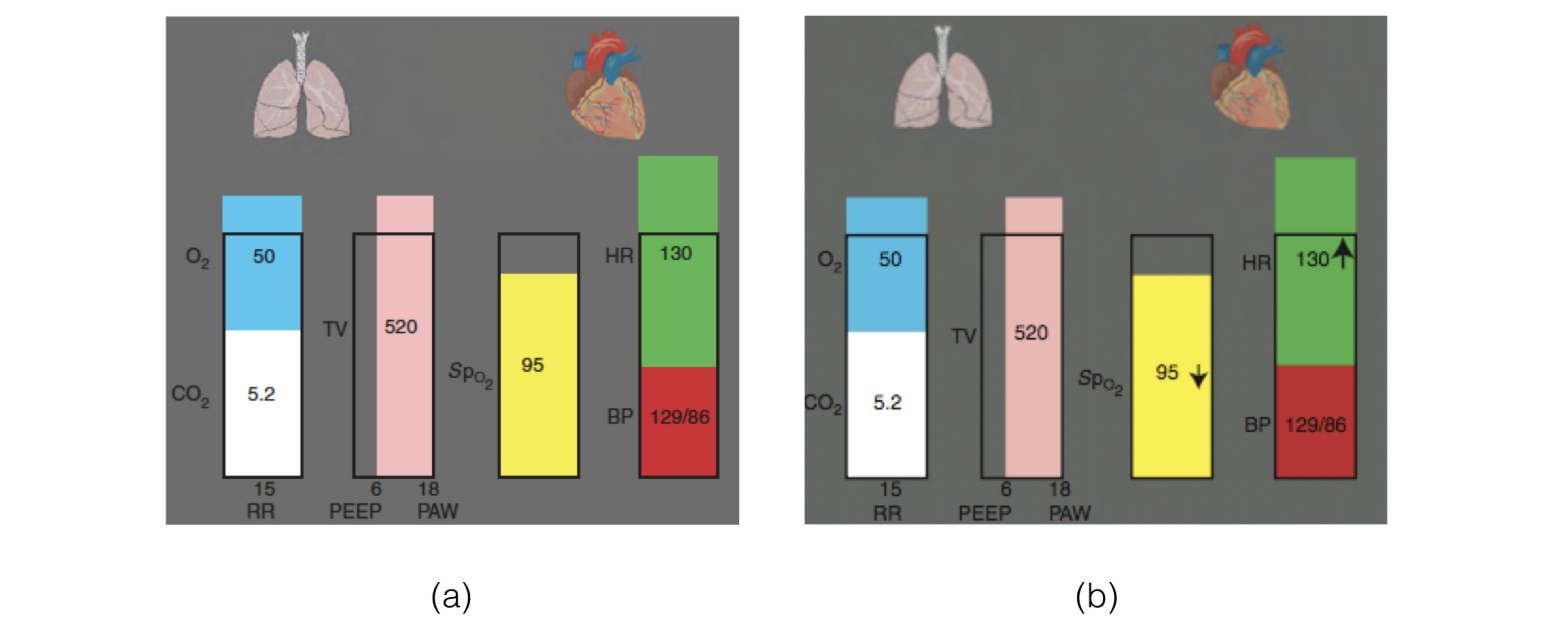
(a) Metaphorical anesthesia interface and (b) Metaphorical interface with trend information (tMAI).

Not all visual metaphors, however, have seen similar success. Zhang and colleagues (2002) [36] introduced an integrated 3-dimensional balloon metaphor, building on the work of Blike and colleagues (2000) [69] with object displays. Zhang and colleagues found mixed results after evaluations, with only 63% of scenarios having shorter detection than scenarios, and situational awareness being improved in 1 of 4 scenarios. Moreover, van Amsterdam and colleagues (2013) from the University Medical Center Groningen, utilized customization features offered by vendor-based medical monitors to construct and evaluate a metaphoric display presented in Figure 9 [10]. They found, however, that visual metaphors did not improve detection or accuracy of anesthesiologists [10].

Finally, while ecologic representations were evaluated for diagnostic accuracy and speed, the challenges surrounding cognitive errors remain only a secondary concern in research involving visual representations. Less than 8 out of 39 of papers analyzed were identified to have measured for cognitive workload [5,7,8,19,22,34,36,41]. Of the 8 papers that measured for cognitive workload, 4 papers used a quantitative measure such as the NASA-TLX score [5,8,22,34]. There are also limitations with the use of NASA-TLX, largely because it is a self-reported method of identifying perceived workload. A total of 3 of the 8 papers were evaluated with critical care clinicians, consequently, incorporating cognitive workload as a passive measure of potential cognitive error remains limited across visual representation research for clinical environments. Significantly, none of metaphoric displays supported analytic functions.

### Tri-Event Parameters

Physiological displays can be designed and developed using 3 consumption efficacy metrics derived for temporal and dynamic data streams [43]. These metrics are termed tri-event temporal parameters namely, trajectory, frequency, and duration of salient events.

**Figure 10:**
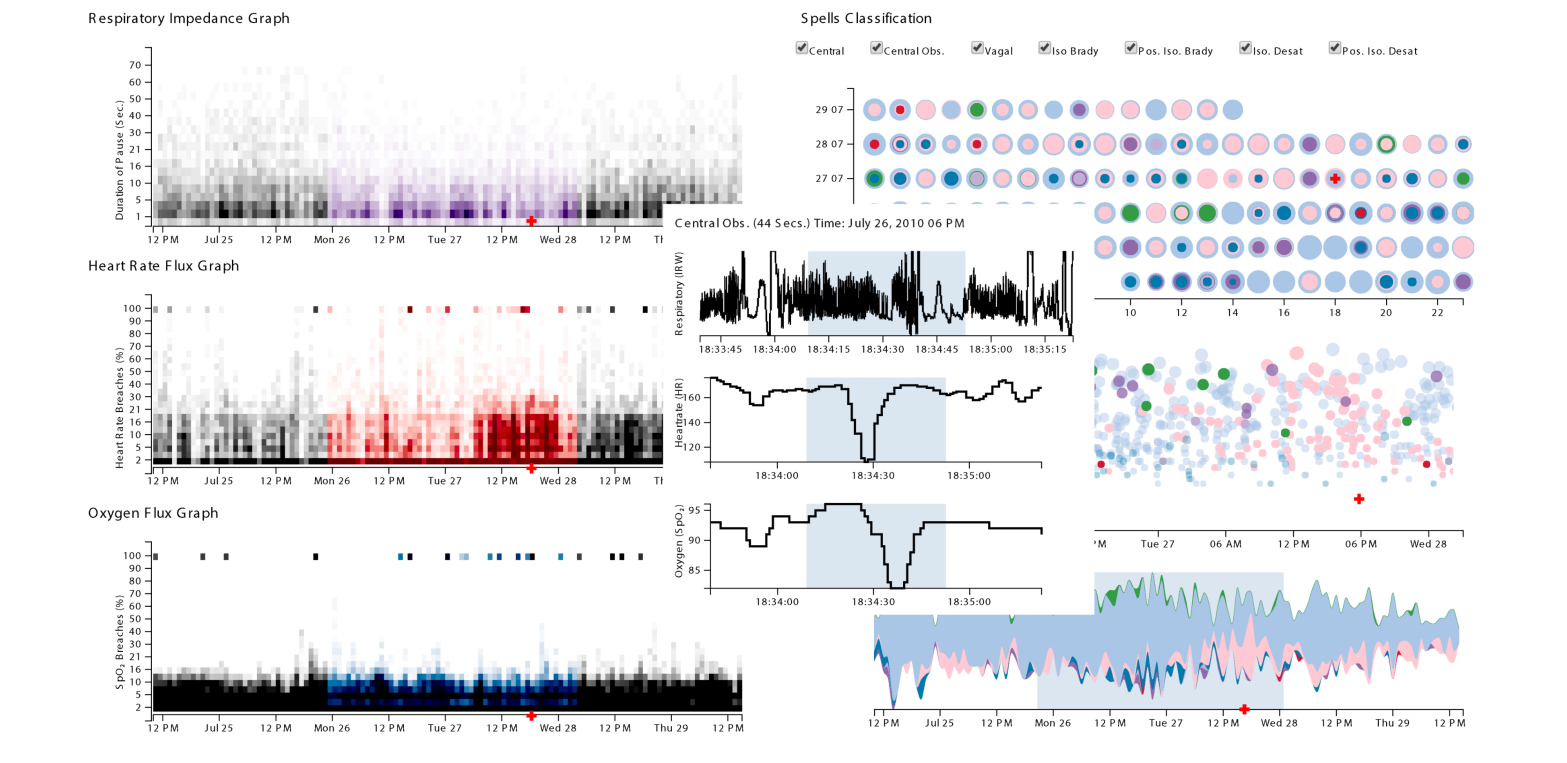
PhysioEx, a coordinated visual analytic tool for exploring clinical events across multiple temporal physiologic data streams.

Among the tri-event parameters, trajectory was found to be the most popular, with 32 of 39 studies incorporating some form of trajectory information. However, longitudinal trajectory was found in only 9 studies, and was rare among displays that were found in anesthesiology but more common in critical care. Displays that incorporated an aspect of the tri-event temporal parameters exclusively adopted trajectory. Nine visual representations were found to have included the duration and frequency metrics. Most of the representations that included duration and frequency used glyphs (n=6) or text (n=5) to communicate episodic information. For instance, PhysioEx [43] uses the river metaphor [72] to illustrate frequency of adverse physiologic events that were analyzed by a real-time algorithm (bottom left, in Figure 10). Text also remains a popular method for communicating discrete events. Law and colleagues found text to be superior to waveform and numeric displays when communicating clinical episodes, even while clinicians reported a preference for graphical displays [73]. Where multiple views were presented, only one representation utilized interactive coordination between independent views [6].

### Conclusion

Visual representations of physiological data have been attempted several times as witnessed by the sheer size of prior work discussed in this paper. Many have shown their potential to improve clinical care, and while largely positive results have been released, there are still concerns as to the efficacy of both in reproducibility as well as translatability to the unit. In particular, methods to identify the accuracy of actions post-treatment to the display remain as concern and open areas for further exploration.

Few clinical visualization papers studied associations of the treatment condition to the accuracy or accrued insight by the user. It was also seen that most studies included detailed study of the time to diagnosis and its accuracy; however, many of these studies included highly controlled scenarios with highly visible graphical distortions. Additionally, few studies used real patient data to evaluate their prototypes. Hence, the frequency of events with clear and distinctive graphical patterns existing across real patient data remains untested. Detection was also another area where studies frequently report positive findings; however, in many cases these differences were marginal and found in narrow statistical ranges. It has yet to be proven whether these statistical significances are relevant in the clinical domain. Exact mechanisms inducing positive effect have yet to be studied within the prototypes studied [63,74].

Visual representations show promise; however, they are plagued with user-preference and interaction challenges. Results spanning two decades continue to show positive influence of graphical representations when they are used in simulated studies [4]. However, many of these studies have not used standardized metrics to test distinct controlled variables, or provide evidence of precisely which features of the graphical displays afford greater comprehension to the consumer. Questions still remain as to its efficacy in clinical practice, where, the availability of all data required by the representations may be limited. There is also the limitation of graphical representation failing to maintain interpretable coherence, when provided incorrect data [2].

Some studies have also demonstrated user involvement as an important factor which may have influenced results, in the design and development of the clinical system [45]. Future studies should focus on clinical validation as a means to identify real-life relevance. Clinical experiments are difficult in lieu of several considerations and their limitations. However, one study by Wachter et al [9], demonstrates that observational studies, although somewhat intrusive, may produce some significant qualitative results. These studies need to be expanded, and clinical trials must ultimately demonstrate their efficacy. Cognitive errors also require additional research effort, specifically by including evaluation methodologies such as the NASA-TLX score to allow end-users to self-report perceived workloads.

Only 7 visualizations were identified to have had some element of interactive selection and filtering functions to support basic analysis tasks. While only one display was identified to support analysis across cohort populations. The general absence of analysis functionalities is an opportunity for enhancing physiologic visualizations. Physiologic data represents a unique subset, due to the dynamic and streaming nature of the data. Application of visual analysis techniques may support novel uses of physiologic visualizations, such as supporting human-driven hypothesis generation tasks.

Finally, research in visual representations should include tri-event parameters as important design considerations to produce designs that communicate episodic information. PhysioEx was seen to incorporate all 3 parameters; however, it was limited to one view per patient [43]. These visual representations can then be used to better assess the influence of tri-event parameters on higher level workflows as well as in the progression of clinical conditions.

## Abbreviations

0: No Change
App: Application
C: Curves
Des: Design
Eval: Evaluation
Exp: Experiment
G: Glyph
MT: Metaphoric display
Neg: Negative
NI: Not included
O: Object
OB: Object-based display
Pos: Positive
Sim: Simulated
T: Text
TB: Tabular display
WF: waveform display
